# Natural history matters: Plastics in estuarine fish and sediments at the mouth of an urban watershed

**DOI:** 10.1371/journal.pone.0229777

**Published:** 2020-03-18

**Authors:** Theresa Sinicrope Talley, Nina Venuti, Rachel Whelan

**Affiliations:** 1 California Sea Grant, Scripps Institution of Oceanography, University of California San Diego, La Jolla, California, United States of America; 2 Environmental and Ocean Sciences, University of San Diego, San Diego, California, United States of America; University of Waikato, NEW ZEALAND

## Abstract

The extent to which small plastics and potentially associated compounds are entering coastal food webs, especially in estuarine systems, is only beginning to be realized. This study examined an estuarine reach at the mouth of urbanized Chollas Creek in San Diego, California to determine: 1) the extent and magnitude of microplastics pollution in estuarine sediments and fish, 2) the extent and magnitude of SVOC contamination in estuarine fish, and 3) whether fish preferentially ingested certain types of microplastics, when compared with the microplastic composition of creekbed sediments. Surface sediments (0–5 cm depth) contained about 10,000 small plastic pieces per m^2^, consisting mostly (90%) of fibers, and hard and soft pieces. Nearly 25% of fish contained small plastics, but prevalence varied with size and between species. Of the 25 types of small plastics found in sediment, fish preferred about 10 types (distinct colors and forms). Several SVOCs, both water soluble and sediment-associated compounds, were found in the two species of fish tested. This study revealed that a species’ natural history may influence contamination levels, and warrants further study to better understand the pathways of plastics and associated contaminants into and throughout coastal food webs, and the potential health risks for small and/or low-trophic level organisms.

## Introduction

Much research conducted over the last two decades has revealed that microplastics (plastic particles <5mm; [1]) are pervasive in marine systems around the world [2,3]. There has been a recent shift to examine the extent and magnitude of microplastics in terrestrial and freshwater systems [1,4], which is important given that significant portions of marine plastic pollution come from land-based sources and rivers, especially urbanized rivers, which are major conduits of debris from land to sea [5–13].

Small plastics enter the environment as either primary microplastics, those manufactured as tiny pieces such as microbeads, or secondary microplastics, those that form from the breakdown of larger plastic items [14,15]. These microplastics are of concern because they are ubiquitous, easily transported in flowing water, and harmful to biota [14,15]. Plastics may be inadvertently consumed by organisms, such as filter feeders (e.g., clams) or deposit feeders (e.g, earthworms) that feed relatively non-discriminately on appropriately sized prey, or intentionally consumed, such as when organisms cannot differentiate plastics from prey (e.g., fish, lobster) [14,15,16]. While some microplastics may pass through an organism’s digestive tract, there are many risks associated with consumption. Consumption of plastics may reduce overall food intake, and therefore fitness, and may cause physical damage to an organism’s digestive tract [15,17]. Further, microplastics can accumulate in the gut or gills of organisms, interfering with important life history processes such as feeding, growth, and reproduction [4,16–19]. The monomers and additives that compose microplastics can be toxic to biota if they leach from their parent plastics into the environment [20–22]. Furthermore, microplastics can sorb toxins such as metals, PCBs, PAHs, and DDT from the aquatic environment [23], and transmit these toxins to organisms [24], causing stress to internal organs, disruptions in normal bodily functions (e.g., enzyme inhibition, endocrine disruption), and reductions in organisms’ abilities to defend themselves against predators and other threats [4,21,25–29]. Microplastics have been found to transfer between trophic levels [30–33], and thus may pose health risks to humans via consumption of contaminated seafood (e.g., [34,35], though the impacts of microplastics on human health remain largely unknown [21,36].

Additives commonly used in plastics manufacturing, such as phthalates, bisphenol A, PBDEs, PCBs, PAHs, and DDT [37–39] are all semi-volatile organic compounds (SVOCs). SVOCs are of concern for humans and wildlife because they are endocrine disrupting chemicals (EDCs) that have been linked with neurological, reproductive, metabolic, and behavioral abnormalities, as well as increased incidences of some forms of cancer [40–42]. SVOCs are susceptible to leaching out of plastics into the environment and also resorbing to microplastics once present in the environment due to their hydrophobic properties ([20,26,38,43]. There are also many other sources of SVOCs in the environment, including household cleaning products, cosmetics, and pesticides [37,39]. Microplastics’ role as a conduit for SVOCs into coastal food webs may be relatively unimportant when compared with other vectors, such as contaminated water, prey, or sediments [38,44]. Microplastics can, however, facilitate the accumulation of SVOCs in organismal tissues [27,45], making it important to understand the types, fates, and effects of small plastics and associated contaminants in coastal watersheds in order to develop natural and social science-based solutions to marine debris and declining watershed health [1].

This study, therefore, contributes to the growing body of research on microplastics upstream from marine ecosystems by examining a brackish reach of Chollas Creek, an urbanized creek that connects mid-city San Diego with San Diego Bay, to determine: 1) the extent and magnitude of microplastics pollution in estuarine sediments and fish, 2) the extent and magnitude of SVOC contamination in estuarine fish, and 3) whether fish preferentially ingested certain types of microplastics, when compared with the microplastic composition of creekbed sediments.

## Materials and methods

### Study location

**Fig 1:**
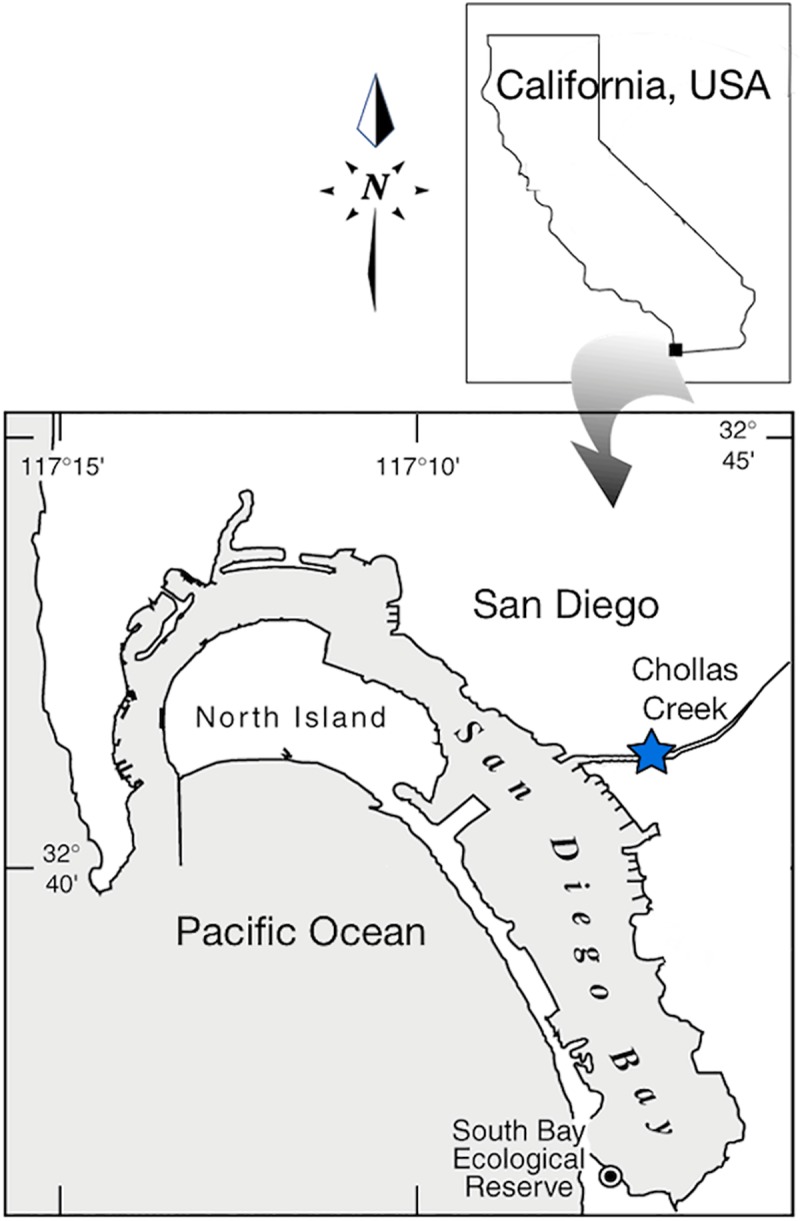
Location of study site. Study site located in lower Chollas Creek near the mouth with San Diego Bay, California, USA.

The Chollas Creek subwatershed (Fig 1) is considered one of the most impaired waterbodies in San Diego County, due largely to nonpoint source pollution in runoff and significant inputs of trash during both dry and wet seasons [46–49]. Chollas Creek originates from, and runs through, a densely populated, urban section of the county [50] and empties at one of coastal San Diego’s most polluted runoff sites [51] in San Diego Bay, reported to be the second most polluted bay in the country [5]. In June 2015, sediments and fish were sampled along a 250-m long reach of tidal brackish Chollas Creek, located about 1.5 km upstream of the mouth (Latitude: 32.6953° N, Longitude:-117.1230° W; Fig 1).

### Sample collection

Estuarine sediments were collected at low tide throughout the reach of creek, in nine 10-cm diameter x 5-cm depth cores (393 cu cm). Cores were placed in clean, airtight zip-top bags, and frozen until analysis in the lab.

Common wetland fish were trapped using metal minnow traps baited with cat food placed in nylon sleeves (to prevent fish from consuming it) and set throughout the reach. Three species were captured: the native marsh residents California killifish (*Fundulus parvipinnis*; n = 68) and longjaw mudsucker (*Gillichthys mirabilis*; n = 4), and the introduced sailfin molly (*Poecilia latipinna*; n = 82). In the field, all fish collected for gut analysis were placed in zip-top bags (one bag per trap). Additionally, two composite samples (7 California killifish and 8 sailfin molly) were collected and immediately placed into clean glass jars for analysis of SVOCs. Only four longjaw mudsucker individuals were captured, so all were used for gut analysis. All fish were frozen until analysis. A protocol of the AVMA Panel on Euthanasia [52], rapid chilling to euthanize warm-water fish, was followed to humanely euthanize the fishes. Use of AVMA protocols for euthanasia of these wetland fishes was approved by the University of San Diego Institutional Animal Care and Use Committee (IACUC), and IACUC training was received prior to the start of this research (report 3382698, TST). All collections were made in accordance with California Dept of Fish and Wildlife (Permit SC-5295), and no permissions were needed to access the field site, which is a City of San Diego public access point.

### Sample processing and analysis

Plastics were sorted from sediments by placing a single layer of sediment at a time into a Petri dish (about 1 tablespoon or 15 cu cm) along with a squeeze of milliQ water to slightly liquefy the moist sediment. The dish with mud was systematically examined at 25-45x power using a dissecting microscope; sorting of each dish took no more than 15 minutes. Particles that were clearly of anthropogenic origin, as determined by the shape and/or color of each particle (e.g., spherical microbeads, fibers with smooth surfaces and homogeneous thicknesses; often bright colors that stood out from the rest of the sample) were sorted out of the sample, classified according to type (film, hard piece, soft piece, fiber) and color, and then counted and measured for maximum length. Particles that were not clearly of anthropogenic origin were examined using a compound microscope to check for lack of cell structure. Any particles that remained of uncertain origin after being investigated through a compound microscope were excluded from the analysis. Anthropogenic particles removed from sediments ranged from 0.5-5mm in size.

Fish used for gut analysis were thawed, measured, weighed, and sexed in the lab. A ventral, longitudinal incision and two perpendicular ventral incisions (anterior and posterior) were made in each fish to expose the intact guts, and then the fish was placed in a Petri dish under a dissecting microscope to complete the dissection and removal of gut contents. All contents were removed from inside the fish gut systematically as the gut was opened and analyzed a small section at a time, for a total of approximately 15 minutes of exposure time. Only materials drawn out of the gut were identified (or described) and counted. As with sediment samples, anthropogenic particles were identified by shape, color, and/or lack of cell structure, categorized by type (film, hard piece, soft piece, fiber) and color, and then counted and measured for maximum length. Anthropogenic particles found in fish guts ranged from 0.05–6 mm in size, but only those particles <5 mm were included in analyses (one 6 mm-long red fiber was removed). Organic items found in fish guts were also counted, or, in the case of items not feasibly counted (e.g., sand grains, organic debris, filamentous algae), presence in the gut was noted. Ten out of the 149 fish sampled had empty guts and were therefore excluded from further analyses.

For SVOC analyses, composite fish samples were analyzed for 67 SVOCs by a local analytical facility (Enviromatrix Analytical, Inc.) using EPA Method 8270C [53].

### Limitations of microscopy

It is important to note that because samples were only examined for microplastics via microscopy, and not run through a spectroscope to chemically verify polymer types and particle counts, it is possible that the abundances of microplastics reported herein are either over- or underestimates of actual numbers, as both false positives and failures to detect very small plastic particles are relatively common when relying on microscopy to identify microplastics [54–56].

### Contaminant control in sample processing

At the time this study was conducted, the risk of sample contamination from airborne plastics was just beginning to be realized [57,58], but see [59], and protocols to control for such contamination followed soon after (e.g., [35,60,61]), though such QA/QC protocols have not yet been standardized, and debate remains about how best to control and account for sample contamination [56,62]. Therefore, a post-hoc control of environmental plastics contamination was conducted using three trials separated in time on 20 June, 20 July and 19 August 2016 to determine average levels of contamination in the lab used during the study. During each trial, six clean Petri dishes were set out for 15 minutes on the lab countertops. Three or four people were present in the lab each time (during sample sorting in 2015, two or three people were present at any one time). At 15 minutes, dishes were covered with clean, clear lids and examined for particle settlement using a dissecting microscope. Only fibers were found at an average of 0.5±0.3, 0.5±0.2 and 0.5±0.3 fibers per dish for the June, July and August trials, respectively (grand average = 0.5±0.0 fibers per dish per 15-minute time period). Fiber contamination for each fiber color (type) was then calculated using the following steps: the average number of fibers per dish (0.5) was multiplied by the number of dishes likely sorted for each sediment sample core (393 cu cm core / 15 cu cm spoonful per dish = ~26 dishes per core) for an estimated total of 13.1 fibers contaminating each sample core. Since the color of fibers causing contamination in 2015 could not be determined after the fact, the estimate of 13.1 fibers per core was divided by the seven fiber color categories for an estimate of 1.87 fibers contaminating each fiber color category. This value (1.87 fibers) was then subtracted from each fiber color category of each core before analyses and summary statistics were calculated. If the result of the subtraction was a negative number, then a 0 value was assigned.

As discussed above, fish gut contents were analyzed little by little, as the guts were drawn out of the fishes’ bodies, resulting in only ~15 minutes of total exposure (and therefore, low risk of contamination). Since the post-hoc estimated lab contamination rates were ≤0.5 fibers per 15-minute sample (and 0.5 fibers per dish divided by the one to four fiber color categories found in the fish samples equals 0.125–0.5 fibers contaminating each fiber color category), a contaminant correction was not used for fish samples, but it is acknowledged that fiber counts may be slight overestimates.

### Data analyses

Descriptive statistics of sediment microplastics (average of all cores) and fish gut contents (average of small plastics and prey items for each species) were calculated to summarize findings. The concentrations of SVOCs, if present in at least one composite sample (at least one of the species), are reported. Fish diet preference was explored using Manly’s alpha [63], which compares the abundances of the types of plastics found in the environment and consumed by the fish. Differences in size and sex ratios of all fish sampled to those that had consumed plastics were tested using t-tests (size variables) and Chi Square (sex ratios) in JMP 12.

## Results

### Microplastics in sediment

**Fig 2:**
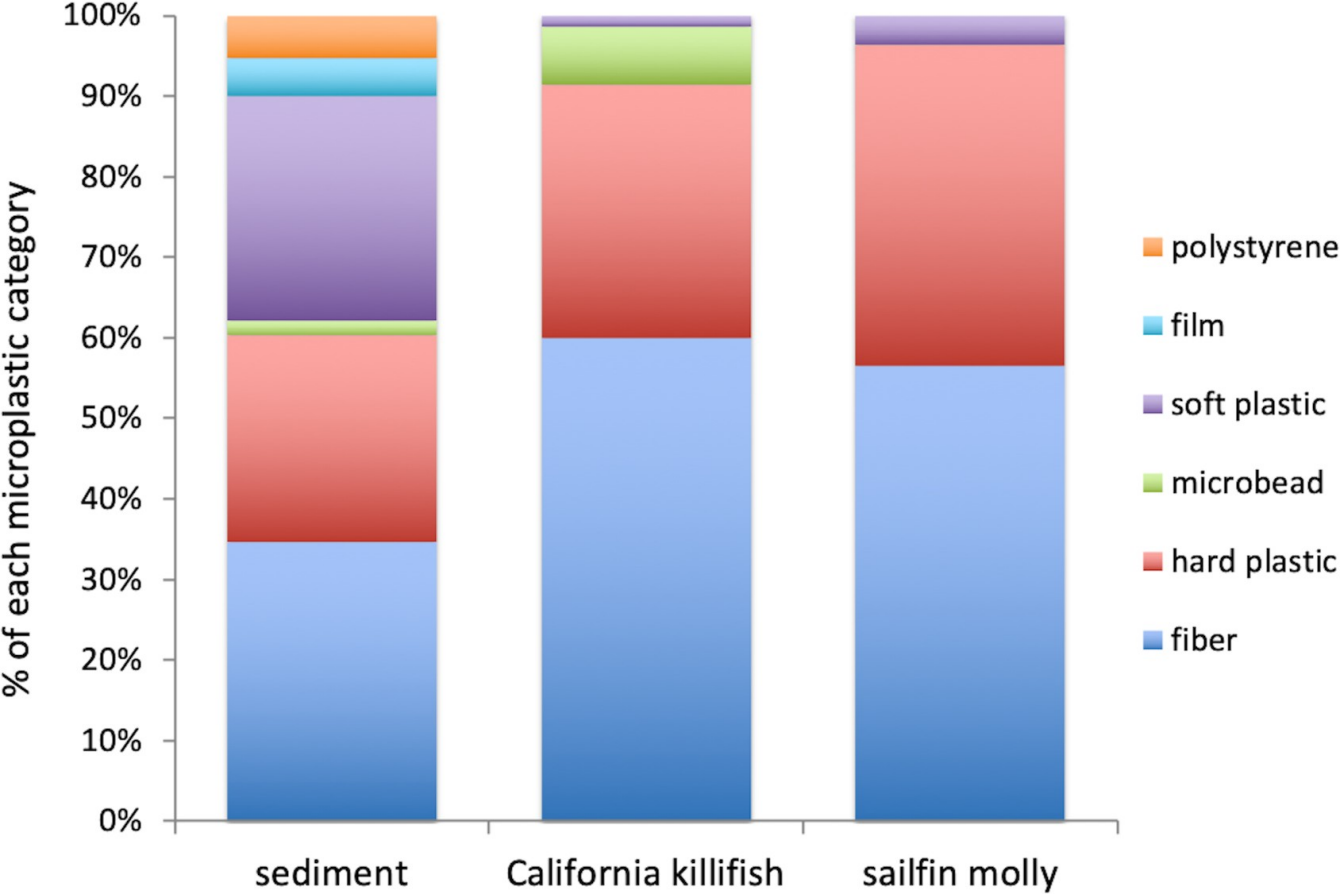
Sediment and fish microplastics. Relative abundance and composition of microplastics found in sediments (0–5 cm depth) and the guts of two common wetland resident fish in lower Chollas Creek. N = 9 soils, 7 California killifish, 23 sailfin molly. Data are from June 2015.

All sediment cores collected contained microplastics; the average abundance (±1SE) was 9,544±1,413 pieces m^-2^ and average lengths (±1SE) of small plastics ranged from 0.5±0.1 to 4.9±0.1 mm. Common categories of plastics were film pieces, polystyrene pieces, soft pieces, hard pieces, microbeads, and synthetic fibers (Fig 2, Table 1), with synthetic fibers, hard pieces, and soft pieces together making up 90% of the fragments found in sediments. Nearly half of all fragments were white or clear (Table 1).

**Table 1:** Microplastics abundance in sediments and fish.

Type of microplastic	sediment	California killifsh	sailfin molly	California killifsh	sailfin molly
n =	9 cores	7 individuals	23 individuals	7 individuals	23 individuals
	**Abundance (no. m^-2^)**	**Abundance (no. gut^-1^)**		**Manly's alpha (α ≥ 0.040)**	
	**Avg±1 SE**	**Avg ±1 SE**	**Avg± 1 SE**	**Avg± 1 SE**	**Avg± 1 SE**
**Polystyrene piece**	396 ±105	0	0	0	0
**Rubber or foam piece**	28± 28	0	0	0	0
**Film**					
clear or white	283±76	0	0	0	0
silver or gray	42±30	0	0	0	0
**Soft plastic pieces**	** **	** **			
clear or white	481±108	0	0	0	0
green	269±113	0	0	0	0
blue	467±192	0.14± 0.14	0.13± 0.13	0.004 ± 0.001	0.004± 0.002
red	1062±389	0	0	0	0
yellow	410±137	0	0	0	0
orange	99±59	0	0	0	0
**Hard plastic pieces**	** **	** **	** **		** **
clear or white	524±212	0	0.04±0.08	0	0
green	368±157	0.14± 0.14	0.04±0.08	0.004± 0.004	0.002±0.002
pink	28±28	0	0	0	0
blue	311±150	0.29±0.18	0.65±0.52	**0.100±0.076**	**0.181±0.178**
red	424±133	0.14±0.14	0.17±0.19	0.002±0.002	**0.066±0.049**
yellow	1019±376	0.14±0.14	0.13±0.13	**0.143±0.143**	**0.051±0.048**
black	14±14	0	0	0	0
silver	14±14	0	0	0	0
orange	14±14	0	0.04± 0.08	0	**0.044± 0.044**
microbead	212±124	0.29± 0.18	0	**0.099±0.099**	0
**Fibers**					
clear or white	2923±961	0.57± 0.43	0	**0.145±0.143**	0
black	32±21	0	0.39±0.36	0	**0.143±0.064**
blue	44±22	0.43±0.30	0.43±0.25	**0.173±0.141**	**0.227±0.080**
green	16±16	0.14±0.14	0.13±0.13	**0.181±0.138**	**0.143±0.078**
red	32±28	0.57±0.30	0.22±0.20	**0.153±0.075**	**0.133±0.131**
**Total per m**^**2**^ **or gut:**	9544±1413	2.86±1.37	2.43±0.68		

Abundance of microplastics in surface sediments and the guts of common marsh resident fish, and Manly’s alpha where a ≥0.040 (in bold) indicates a dietary selective preference for microplastics compared to what were available in the environment. Only individuals with microplastics present in the gut were included in this summary. Samples were collected from lower Chollas Creek, San Diego, California, USA during June 2015.

### Fish and microplastics

#### Prey and non-plastics in guts

Half or more of the California killifish, longjaw mudsucker, and sailfin molly collected had fed on sand or silt particles, and filamentous green algae (Table 2). Roughly 10% of both California killifish and sailfin molly also contained red algal filaments (Table 2). California killifish, primarily an invertebrate predator [64], contained remnants of small crustaceans (e.g., exoskeleton pieces, amphipod appendages), scales, unidentifiable fishes and insects, snails, tubificid oligochaetes, nematodes, and a sea cucumber (Table 2). The sailfin molly, although predominantly an herbivore [65], also commonly ingested tubificid oligochaetes and nematodes, and to a lesser extent, crustaceans and snails (Table 2). The predatory longjaw mudsucker contained scales, a digested fish, and nematodes (Table 2).

**Table 2:** Non-plastic fish gut contents.

Gut content items	California killifish	sailfin molly	longjawed mudsucker
n (number of individuals) =	61	74	4
**Items that could not be counted**	**Avg**± **1 SE**	**Avg**± **1 SE**	**Avg** ± **1 SE**
** **	**% of fish with items present**		
sand or silt	48%	99%	50%
scales	5%	0%	25%
unknown exoskeleton pieces	53%	1%	0%
unknown amphipod or shrimp pieces	2%	0%	0%
unknown decapod pieces	0%	1%	0%
unknown organics or digested pieces	18%	0%	0%
green filamentous algae	75%	85%	50%
red filamentous algae	10%	11%	0%
**Enumerated items**	**Avg ±1SE**	**Avg± 1SE**	**Avg ± 1SE**
	**(no. gut**^**-1**^**)**	**(no. gut**^**-1**^**)**	**(no. gut**^**-1**^**)**
snails (*Barleeia californica*, *Assiminea californica*)	0.49±0.40	0.04±0.03	0
tubificid oligochaetes, nematodes	1.66±0.28	0.12±0.05	1.75±0.75
unknown whole digested-fish	0.02±0.02	0.01±0.01	0.25±0.25
unknown fish eggs or larvae	0.05±0.05	0	0
unknown insect larvae or adult parts	0.05±0.03	0	0
sea cucumber *(Leptosynapta* sp.)	0.05±0.03	0	0

Abundance of prey and other non-plastics found in the guts of common marsh resident fish from lower Chollas Creek, San Diego, California, USA. Data are from June 2015.

#### Characteristics of plastic eating fish

None of the longjaw mudsucker guts contained plastics, which may have been due to the small sample size of only four individuals. California killifish individuals that had plastics in their guts were, on average, 25% longer and 79% heavier than those free of plastics (p≤0.03, Table 3). In fact, 24% of individuals that were 5.3 cm total length and weighed 2.6 g weight (the average total length and weight) and greater had plastics in their guts, while 3% of individuals shorter and lighter than the average had plastics. The ratio of males to females (to unknown sex) was similar between fish with and without gut plastics (Table 3). Neither size nor sex of sailfin molly individuals differed between those that contained plastics and those that did not (Table 3).

**Table 3:** Comparison of fish with and without plastics.

	**California killifish**				
	**Fish without plastics**	**Fish with plastics**	**t-test/Chi square results**		
n =	61	7			
**Variable**	**Avg** ±**1 SE**	**Avg**± **1 SE**	**P**	**t/ Chi sq**	**df**
standard length (cm)	4.34±0.16	5.50±0.32	0.003	2.52	59
total length (cm)	5.12±0.18	6.40±0.40	0.005	2.41	59
weight (g)	2.35±0.27	4.20±0.32	0.005	2.25	59
sex: female / male / unknown	24/23/6	3/3/1	0.571	1.13	2
	**sailfin molly**				
	**Fish without plastics**	**Fish with plastics**	**t-test/Chi square results**		
n =	74	23			
**Variable**	**Avg**± **1 SE**	**Avg**± **1 SE**	**P**	**t/ Chi sq**	**df**
standard length (cm)	4.05±0.12	4.15±0.29	0.655	0.55	72
total length (cm)	4.96±0.14	5.08±0.34	0.624	0.58	72
weight (g)	2.05±0.19	2.39±0.38	0.466	1.04	72
sex: female / male / unknown	28/21/2	15/7/1	0.677	0.78	2

Comparison of fish that had and did not have microplastics in their guts with all fish analyzed in this study. Results of t-tests (fish morphological variables) and Chi square (sex ratios) are shown.

#### Microplastics in fish guts

Almost one quarter of fish examined contained small plastics, with 12% of California killifish (7 of 61) and 32% of sailfin molly (24 of 75) having consumed plastic (Table 3). Of the 25 types of plastic available in the environment, the California killifish and sailfin molly each consumed 10–11 different types of plastic items, mostly consisting of fibers and hard pieces (Fig 2, Table 1) that ranged in length from 0.05 to 5 mm.

Of the 10–11 types of small plastics that were consumed by the fishes, 7–8 types were selectively eaten (Manly’s alpha ≥0.040; Table 1), meaning the fishes’ guts contained higher proportions of these items than were found in the environment (Fig 2). The items the fishes selected included blue, yellow, orange and/or red hard plastic pieces, and blue, green, red, black, white and/or clear fibers (Table 1). California killifish additionally preferentially consumed microbeads (Fig 2, Table 1).

### SVOCs in fish

**Fig 3:**
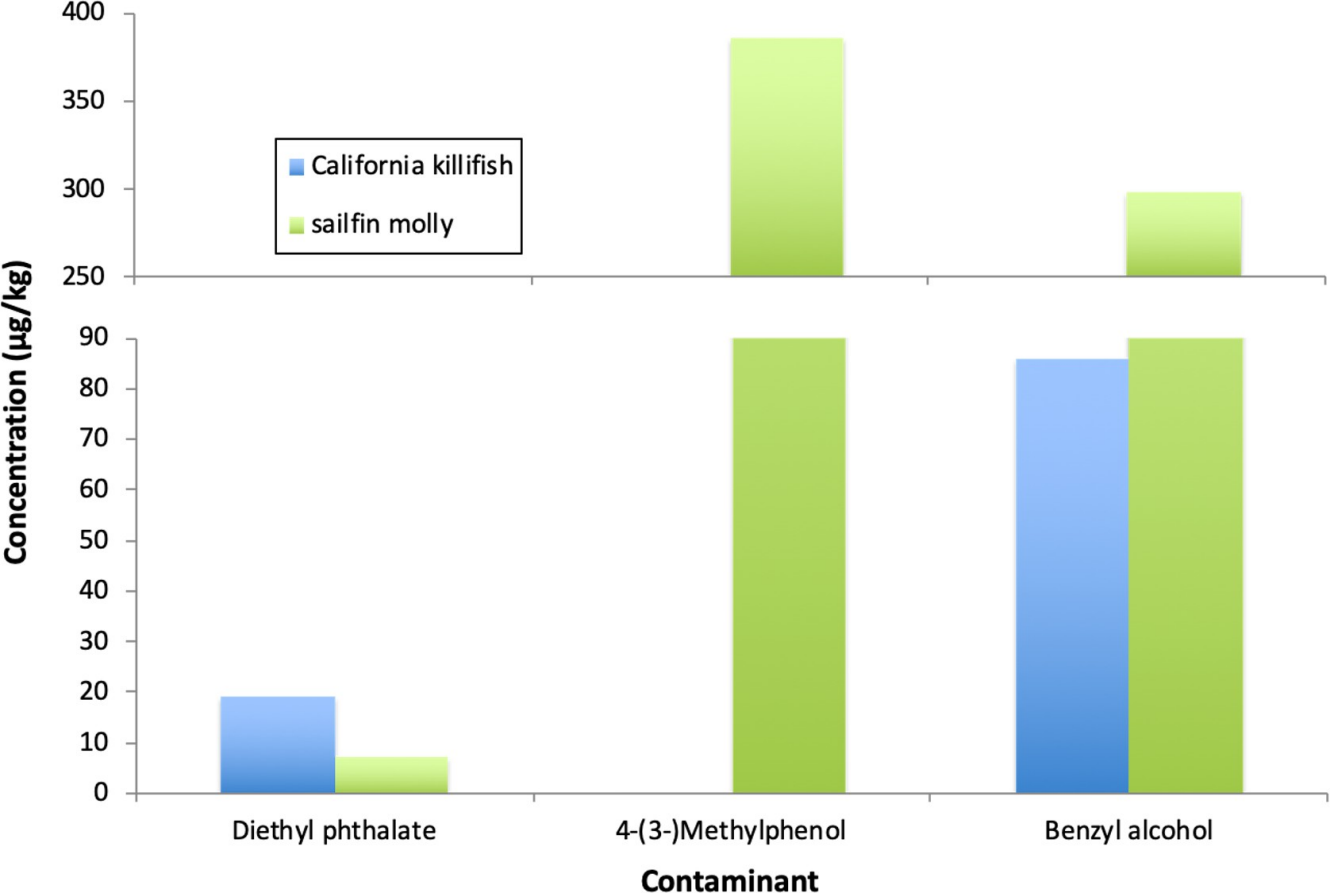
SVOCs in fish. Semi-volatile organic compounds found in wetland resident fish in lower Chollas Creek, San Diego, California, USA. N = 1 composite sample per species (3 California killifish and 2 sailfin molly individuals). Data are from June 2015.

Three SVOCs of 67 tested were found in the tissues of these species. Both the California killifish and sailfin molly contained diethyl phthalate and benzyl alcohol, and the sailfin molly additionally contained 4-(3-) methylphenol (Fig 3). The sources of these compounds in this creek are uncertain. The phthalate is strictly a synthetic compound, while benzyl alcohol and 4-(3-) methylphenol have both synthetic and natural, albeit localized, sources [66,67,68]. All three have common industrial applications as additives in plastics, solvents, antiseptics, preservatives, pesticides, and/or additives in cosmetics and perfumes [66–71].

## Discussion

### Microplastics in upstream sediments

Examining the extent and magnitude of plastics pollution upstream of marine systems provides insights into the land-based sources and transport mechanisms of marine debris, and the potential dynamics and impacts of these pollutants throughout watersheds. This study, particularly, provides key insight into microplastics loads and dynamics in the Chollas Creek subwatershed, as it revealed densities of microplastics at the mouth of urbanized Chollas Creek that were 70–500 times greater (~10,000 pieces/m^2^) than those concurrently found in San Diego bay sediments (avg. of 140 pieces/m^2^) and continental shelf sediments (avg. of ≤20 pieces/m^2^) [10,72]. In contrast, the microplastics densities found at the mouth of Chollas Creek were lower than those found further upstream; around the same time this study was conducted, an average of 13,000 microplastic pieces/m^2^ was found in a freshwater riparian reach of Chollas Creek about 3 km upstream from the study site, and averages of 18,000 and 46,000 pieces/m^2^ were found 5.5–6 km farther upstream in two seasonal tributaries [73]. This suggests heavy inputs from the land uses surrounding and upstream of the creek mouth region.

As stated previously, this subwatershed is highly urbanized, replete with land uses and activities commonly linked to trash inputs, including industrial and high-density residential areas, a large homeless population, and illegal dumping [47,50,74], all crisscrossed by a dense network of roads, which are themselves linked to large inputs of debris and contaminants [10,75]. The abundance of large trash and debris in this region undoubtedly contributes to the accumulation of microplastics by trapping and transporting microplastic particles, and by degrading into small plastic pieces, especially given the sun exposure in this area [76–78]. Large plastics (0.5–50 cm) concurrently found in this and nearby subwatersheds [73,74,79] commonly consisted of types of plastics similar to those observed in this microplastics study, namely hard and soft plastics (e.g., whole and broken containers, flexible packaging), film plastics (e.g., bags, wrappers), synthetic fibers (e.g., clothing, blankets, furniture stuffing), and foamed plastics (polystyrene, rubber), suggesting that the breakdown of larger trash items may be a potentially significant source of microplastics in this watershed. Additionally, microplastics (≤0.5 cm) found in the upstream reaches of this same subwatershed (3, 5.5, 6 km upstream) were similar to those found in the mouth region, namely hard and soft pieces, film, fibers, and microbeads [73], suggesting that sources, accumulations, and, potentially, transport of both primary and secondary microplastics exist throughout the watershed. While it remains important to study the transport and fate of microplastics in bays and oceans [10,12,80], the impacts of these high microplastics densities in upstream seasonal creeks also need to be explored [1,4,81].

### Natural history and contamination risk in fish

**Fig 4:**
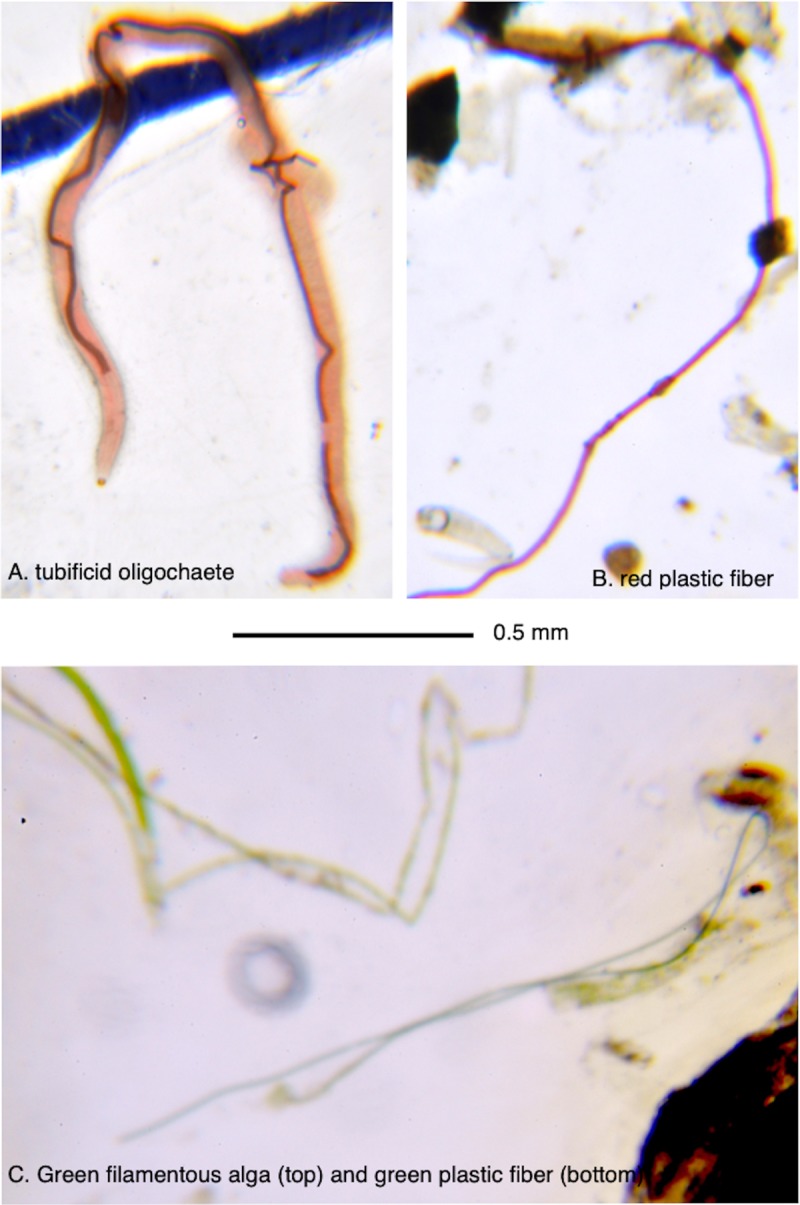
Plastics and food in fish guts. Similar looking microplastic fibers and prey items in guts of wetland resident fish. Contents of California killifish guts included (A) a tubificid oligochaete and (B) a red plastic fiber. Contents of sailfin molly guts included (C) a filament of green algae (top) and a green plastic fiber (bottom). Scale shown applies to all photos.

The extent that plastics and their associated contaminants impact food webs depends upon the types of plastics and contaminants present [21], the environment [82], and the natural history of the organisms (e.g., [83]), including feeding behavior, and changes associated with ontogeny and sex [16,84–88]. While the California killifish is primarily a predator and the sailfin molly is primarily an herbivore, both, as with other estuarine species, intensively forage on the substratum for food items and inadvertently ingest detritus and sediment [86,89]. The substratum is also where dense small plastics and contaminants accumulate, putting these species at particularly high risk of contamination [15]. Omnivorous fishes have also been observed to have four to six times higher abundances of gut plastics than more selective herbivores or predators [88]. Both the predatory and herbivorous estuarine fishes in this study selectively fed on many of the small anthropogenic particles found in their guts, including all colors of fibers, blue and warm colors of hard pieces, and, in the case of California killifish, microbeads. Anecdotally, these items often resembled prey, with similar morphologies observed between fish eggs and microbeads, and between synthetic fibers and filamentous algae, oligochaetes, and nematodes (e.g., Fig 4), further raising the concern that fish and other animals may mistake plastics as food due to similar visual or olfactory cues [90,91]. The likelihood of plastics ingestion or the ability to pass plastics may change throughout the life of an organism, as revealed by the higher incidence of plastics in the guts of larger (older) California killifish individuals during this study. This is consistent with other ontogenetic dietary shifts observed in California killifish, such as changes in prey types, prey sizes, prey abundance, and microhabitat use with time [85,86]. These fish may, in turn, be important vectors for transferring small plastics and contaminants to the broader coastal food web given their abundance, their roles in connecting intertidal with both subtidal and terrestrial ecosystems, and their roles as forage fish for many species [92–95]. While examples of the transfer of plastics between trophic levels are on the rise [30–33], needed is a better understanding of the mechanisms underlying the pathways of microplastics into and through food webs, and the subsequent consequences for these food webs, so that outcomes of contamination may be better predicted.

As with the patterns of plastics ingestion, contamination by SVOCs varied with fish species. Diethyl phthalate, a water-insoluble, sediment-penetrating compound [66], was found in both the California killifish and sailfin molly, but was almost three times higher in the California killifish. Both species ingest sediment while feeding (e.g., Table 3), so an explanation for the higher phthalate concentration in the California killifish is uncertain, but may be linked to diet, with higher abundances of benthic deposit feeders observed in the guts of killifish in this study (i.e., potentially more diethyl phthalate-laden sediment), or, alternatively, may be an artifact of small sample size (n = 1 composite sample). The reasons underlying the presence of 4-(3-) methylphenol and the 3.5-fold greater benzyl alcohol concentration in the sailfin molly, as compared to the California killifish, are also uncertain. These compounds, which are used as solvents, pesticides, antiseptics, anesthetics, and additives in cosmetics and fragrances, are water soluble, have fairly rapid degradation rates in water, and do not tend to accumulate in tissues [67,68]. Again, stomach contents (i.e., water content) or small sample size could explain this observed difference. Although the sources and pathways of exposure are uncertain [37], the presence of these compounds in two samples in this study reveals that transfer of contaminants, even those that are relatively transient, from the environment to food webs is a real risk.

### Health implications of small plastics and SVOCs

The effects of small plastics and SVOC contamination on organisms may be complex (e.g., [82]) and remain largely uncertain, but knowledge of these effects is needed to understand the consequences of exposure to the organisms themselves and to others in the food web, including humans. While plastics are thought to largely pass through the guts of consumers, the extent of retention, degradation, interactions with gut microbiota, and subsequent health effects are only beginning to be realized [96–100], as are the effects of the plastic-associated contaminants (e.g., [100,101]). The acute and chronic effects of the three SVOCs found in this study have been observed on the growth, reproduction, enzyme activity, metabolic activity, respiration, kidney function and/or liver function in animals, while the effects on humans are less well known and are of concern [42,66–68,102–108].

The list of marine life, including seafood items consumed by humans, that contain plastics and associated contaminants is growing [19,21,34,69,109–111]. Therefore, it is not only important to continue to improve our understanding of contaminant dynamics and subsequent health effects throughout food webs, but to translate this information into consumption guidelines. While such guidelines exist to reduce consumer exposure to bioaccumulated contaminants in large, longer-lived, and/or higher trophic level seafood species, this and other recent studies (e.g, [34,97,110,112]) illustrate that smaller and/or lower trophic level fish and shellfish may have hazards of their own linked with plastics and associated compounds.

### Recommendations

This study is one more example in the burgeoning literature on the entry of plastics and associated contaminants into food webs through ingestion. Multiple examples are valuable for documenting the breadth of contexts under which this, and other processes, can occur. Needed now are more efforts to find generalities among examples, such as how trophic level(s), life history strategy, or life style (e.g., benthic, demersal, pelagic) correlate with the incidence of ingestion, and types and levels of contamination. From these patterns, more exploration into the underlying mechanisms is needed so that we may better predict outcomes of contamination (i.e., risk and vulnerability) and effectiveness of proposed solutions.

## Supporting information

S1 Data (DOCX)
