# Supplementary material for: Natural history matters: Plastics in estuarine fish and sediments at the mouth of an urban watershed
Source: PLoS One. 2020 Mar 18;15(3):e0229777. doi: 10.1371/journal.pone.0229777 (PMC7080253; doi:10.1371/journal.pone.0229777)
Supplement: S1 Data — (DOCX) [file pone.0229777.s001.docx]

Re: Talley, Venuti and Whelan

File name: PONE-D-19-24286R1_ftc.docx

Please revise our Data Availability statement to read:

*Data are available at SEANOE Sea Scientific Open Data Publication (seanoe.org), citation:*

*Talley Theresa, Venuti Nina, Whelan Rachel (2015).****Plastics in sediments and fishes at the mouth of Chollas Creek, San Diego, USA****. SEANOE.*[*https://doi.org/10.17882/72119*](https://doi.org/10.17882/72119)
